# Are Inconclusive Decisions in Forensic Science as Deficient as They Are Said to Be?

**DOI:** 10.3389/fpsyg.2019.00520

**Published:** 2019-03-19

**Authors:** Alex Biedermann, Silvia Bozza, Franco Taroni, Joëlle Vuille

**Affiliations:** ^1^Faculty of Law, Criminal Justice and Public Administration, School of Criminal Justice, University of Lausanne, Lausanne, Switzerland; ^2^Department of Economics, Ca'Foscari University of Venice, Venice, Italy; ^3^Faculty of Law, University of Fribourg, Fribourg, Switzerland

**Keywords:** forensic science, evidence evaluation, probabilistic inference, decision making, reporting formats

## Abstract

Many quarters of forensic science use reporting formats such as “identification,” “inconclusive,” and “exclusion.” These types of conclusions express opinions as to whether or not a particular person or object is the source of the material or traces of unknown source that is of interest in a given case. Rendering an “inconclusive” conclusion is sometimes criticized as being inadequate because—supposedly—it does not provide recipients of expert information with helpful directions. In this paper, we critically examine this claim using decision theory. We present and defend the viewpoint according to which deciding to render an “inconclusive” conclusion is, on a formal account, not as inadequate as may commonly be thought. Using elements of decision theory from existing accounts on the topic, we show that inconclusive conclusions can actually be viable alternatives with respect to other types of conclusions, such as “identification.”

## 1. Introduction

When forensic scientists compare questioned material of unknown source with material from a known source, they are often faced with the question of whether the compared materials come from the same source. For example, a fingerprint examiner may be asked to say whether a given fingermark comes form a particular person of interest. Such conclusions are commonly known as “source identifications” (e.g., DOJ, 2018). On the other hand, when the scientist is convinced that the compared items come from different sources, the conclusion will be that the suspect is excluded as the source of the trace (“source exclusion”). The term “inconclusive” is used when “(…) the examiner is unable to identify or exclude the two impressions as originating from the same source” (DOJ, 2018). Similar terminology is used by practitioners working with various other types of traces, such as marks on fired bullets, shoemarks and even handwriting. The practice of reporting forensic findings in terms of identification, inconclusive and exclusion (hereafter IIE) currently faces a lot of criticism, however. The main criticism is that by providing such conclusions, scientists directly express opinions about propositions of interest (i.e., the proposition of common source), a task that is considered to be above and beyond their area of competence (Champod et al., 2016). Rather than expressing opinions on propositions, scientists should concentrate on the value of their findings—i.e., the results of their comparative examinations—and provide an assessment of the extent to which those findings allow one to discriminate between the various propositions of interest (Champod and Evett, 2001). In the latter—probabilistic—perspective, scientists help *others*, i.e., the recipients of expert information, make appropriate use of scientific findings. With other types of traces, especially DNA, this separation of roles, expressing probative value of the findings (scientists) on the one hand vs. reaching conclusions about propositions (fact-finders) on the other, has been in place since the beginnings of the use of DNA in forensic science^1^.

While the IIE terminology is on the decline, mainly in academic circles, it is not destined to disappear. The reason is that, even though scientists may be gradually giving up the IIE terminology when expressing and communicating their findings, legal practitioners will still need to reach conclusions regarding the source of particular items of trace material, based on all available information in a case. Therefore, the IIE terminology, in particular the ways in which the various conclusions (identification, inconclusive, exclusion) are and ought to be understood, is a topic of ongoing interest for research.

The purpose of this paper is to take a closer look at inconclusive conclusions, and how they are (to be) distinguished from identifications and exclusions. The motivation for this focus of inquiry stems from the tendency in some quarters of forensic science to regard “inconclusives” as producing unsatisfying outcomes. Indeed, a report that findings are “inconclusive” in a given case does not offer the recipient of expert information an indication as to which of the propositions of interest is supported by the evidence. This may be fine when the findings are not considered informative (e.g., when the quality is poor and/or there are only few observable features), but is more problematic for findings that have some capacity to point toward one proposition rather than the alternative. In such cases, using the broad term “inconclusive” may give the impression that the findings' value is being lost or squandered in the process. However, as we will show in this paper, the potential loss associated with an erroneous identification or exclusion may be even greater.

The intricacy of both the usefulness and limitations of inconclusive decisions^2^ has recently prompted comments in the forensic literature. Dror and Langenburg (2018), for example, have noted:

“(…) the inconclusive decision is a broad and imprecise decision category for fingerprint examiners, encompassing the range of “almost an exclusion” all the way to “almost an identification.” As such, the weight of the evidence may not be properly conveyed. However, it is an important decision option for analysts to utilize when they actually do not believe the weight of the evidence has surpassed a decision threshold into a definitive categorical decision (i.e., identification or exclusion …).”

Inherent in statements such as these is the view that inconclusive decisions, in some situations, are inadequate. It is important to note, however, that this inadequacy merely reflects a perception: the limited merit or benefit of an inconclusive decision as *judged* by forensic practitioners and researchers, reflecting a widely held mindset in the field. However, this judgment is not based on a formal criterion. It may seem *intuitively* reasonable, but it should be formally justified before it is accepted. This raises a series of intriguing questions, such as “How can one assess the relative merit of a given type of conclusion, in particular inconclusive decisions, using a formal approach?” and “What can a formal analysis of inconclusive decisions tell us about our intuitive attitudes toward this type of conclusion (i.e., can our intuitions be confirmed)?,” in particular “Are inconclusive decisions in forensic science as inadequate as they are said to be?.” In this paper, we will thus seek to approach the question of the appropriateness of inconclusive conclusions in a formal way. We will do so by conducting a comparative analysis between the perception of expert decision making (i.e., decisions on what to conclude following an expert's examination), and a formal analysis of the expert reporting task.

This paper is structured as follows. Section 2 explains the formal method for decision analysis, i.e., decision theory, used throughout this paper, provides definitions and introduces formal notation. In section 3, this analytical apparatus is used to analyse and clarify the conceptual characteristics of inconclusive decisions, and to compare them with those of other types of decisions, especially identifications. Discussion and conclusions are presented in section 4. Here we will present and defend the viewpoint according to which inconclusive decisions, on a formal account, are not as inadequate as may commonly be thought. Actually, as our analyses will show, inconclusive decisions are viable alternatives with respect to other conclusions, such as identification; under certain conditions inconclusive decisions will even be “better” (or, more optimal), in a sense that will be defined later, than identification decisions *despite* a high probability for the proposition according to which the person of interest, rather than an unknown person, is the source of the trace.

## 2. Methods and Definitions

As a starting point for our analysis, we consider expert conclusions as decisions. Given this fundamental starting point, we can proceed further and invoke a formal theory to analyse, characterize and compare decisions: the obvious candidate here is decision theory (e.g., Raiffa, 1968; Lindley, 1985), because it is the reference approach for devising sensible courses of action to be taken by a decision-maker in situations in which adverse decision consequences may be incurred due to uncertainty about the propositions of interest (e.g., Taroni et al., 2005, 2010; Biedermann et al., 2008). In our context of application, examples of propositions are whether the person of interest or an unknown person is the source of a given mark or stain. The fundamental decision problem thus is that, at the time of issuing a conclusion, the examiner does not know whether or not the person of interest truly is the source of the crime mark. Therefore, there is a possibility of incurring, for example, a wrong identification.

We use the following notation for the decision theoretic analyses. We denote by *d*_1_, *d*_2_ and *d*_3_ the three IIE conclusions “identification,” “inconclusive,” and “exclusion,” respectively. Note that one of these conclusions must be given. A choice is to be made among the options *d*_*i*_, *j* = 1, 2, 3, in light of uncertainty about whether the proposition θ_1_, the person of interest is the source of the crime mark, or θ_2_, an unknown person is the source, is true. The propositions θ_*j*_, *j* = 1, 2, also sometimes called states of nature, combine with decisions *d*_*i*_ to lead to decision consequences, denoted *C*_*ij*_. For example, deciding to identify, *d*_1_, when the person of interest is in fact the source of the crime stain, θ_1_, amounts to a correct identification *C*_11_. A further element of our analytical apparatus is the desirability of decision consequences, operationalized here in terms of utility, denoted U(*C*_*ij*_). A decision-maker will face the problem or will need to complete the task of assigning to any decision outcome an expression of desirability that encompasses various dimensions, such as the consequences faced by participants in the proceedings and society at large, as well as societal and systemic values. For the purpose of our analysis, we will measure the desirability of decision outcomes on the standard 0−1 scale, assigning the value 1 to the most desirable consequence(s), and the value 0 to the worst consequence(s). Utility values can be elicited by relying on a procedure described, for example, by Lindley (1985, 2006) that involves coherent comparisons with reference consequences for which the decision-maker has agreed utility values (e.g., 0 and 1). Obviously, the decision-maker is free to choose different reference consequences to build the utility function, and also different scales.

## 3. Analyses and Results

### 3.1. Preliminaries

In discourses about decisions, especially decision analysis and decision-making, it is important to be rigorous in the use of terminology^3^. It is not uncommon, for example, to encounter assertions such as “the utility of this decision is low” or “we seek to maximize utility.” Such assertions are unsound in a classic decision-theoretic sense. Before proceeding, we thus clarify two points. First, following the definition given in section 2, utility does not characterize a decision, but a decision *consequence* (or, outcome). The latter is an outcome resulting from a specific action taken when a given event (or, state of nature) is true. Second, in decision theory, decisions are characterized in terms of *expected utility*. Since there is uncertainty about the state of nature, at the time of making a decision, one cannot know which consequence (with associated utility) will be obtained^4^. At best, thus, only the *expectation* in terms of utility for a given decision can be given, by combining uncertainty about states of nature (quantified in terms of probability) and desirability of possible consequences (expressed in terms of utility). It follows from this that, when there is uncertainty about the state of nature (i.e., and hence what outcome will be obtained), one cannot maximize utility as one could do whenever the state of nature is known, by simply taking the decision to which is associated the larger utility; at best one can only sort out the decision which has the highest *expected utility*. In the next sections, we will rely on expected utility to analyse and compare rival decisions. This may sound technical, but we will show that with suitable choices of the measuring scales and of a pair of consequences that are treated as benchmarks, the proposed framework simplifies considerably and becomes suitable for forensic purposes (or forensic science applications) by limiting the number of choices to be made by the decision-maker during the elicitation of the utility function. In fact, it will be shown that point value assessments for the various components of the analysis are not necessarily required because assessments can also be considered in a qualitative and comparative way.

### 3.2. Analysing and Comparing Rival Decisions

We now consider each of the three IIE conclusions in turn and analyse their properties from a decision theoretic viewpoint.

- *Identification*: The consequences of deciding “identification,” *d*_1_, can either be (i) a correct identification *C*_11_ (which occurs when θ_1_ is true, i.e., the person of interest is the source of the crime stain), or (ii) a false identification *C*_12_ (which occurs when θ_2_ is true, i.e., an unknown person is the source). It is safe to assume that, generally, a correct identification *C*_11_ is the overall most desirable outcome^5^, whereas a false identification *C*_12_ is the overall worst (i.e., most undesirable) outcome. According to the chosen 0−1 scale, and following the approach according to which the best and worst consequences are taken as benchmarks, we thus assign the utility 1 to a correct identification, and the utility 0 to a false identification. Now, if we weigh each of these utilities with the probability of occurrence of the respective consequence^6^, i.e., we compute the expected utility EU of the identification decision *d*_1_, we find that:
  $$\begin{array}{l} \text{EU}\left(d_{1}\right)=\underset{1}{\underset{︸}{\text{U}\left(C_{11}\right)}}\mathrm{Pr}\left(\theta_{1}|I\right)+\underset{0}{\underset{︸}{\text{U}\left(C_{12}\right)}}\mathrm{Pr}\left(\theta_{2}|I\right)\\ \;=\mathrm{Pr}\left(\theta_{1}|I\right). \end{array}$$
  In short, this result means that, given the so-built utility function, the expected utility of an identification decision is directly proportional to the probability that the person of interest is the source of the crime stain. Or, stated otherwise, the higher the decision-maker's belief in the truth of the proposition that the person of interest is the source of the crime stain, the higher the decision-maker's expected utility for the decision to identify the person of interest (see also discussion in Biedermann et al., 2008).
- *Inconclusive*: Here we are directed to think about the desirability of two consequences: (1) the consequence *C*_21_, resulting from concluding “inconclusive” *d*_2_ when the person of interest is the source of the mark (θ_1_ is true), and (2) the consequence *C*_22_, resulting from an inconclusive decision *d*_2_ when an unknown person is the source of the crime scene mark (θ_2_ is true). Note that reporting “inconclusive” will suggest to the recipient of expert information that the findings should be considered as neutral. Hence, it may be argued that it makes no difference whether an “inconclusive” is obtained in the event of θ_1_ (i.e., the person of interest is the source) or θ_2_ (i.e., an unknown person is the source), so that the two utilities U(*C*_12_) and U(*C*_22_) can be taken to be equivalent. Under this assumption, the expected utility EU of an inconclusive decision *d*_2_ becomes:
  $$\begin{array}{l} \text{EU}\left(d_{2}\right)=\text{U}\left(C_{21}\right)\mathrm{Pr}\left(\theta_{1}|I\right)+\text{U}\left(C_{22}\right)\mathrm{Pr}\left(\theta_{2}|I\right)\\ \;=\alpha ,\text{ if }\alpha =\text{U}\left(C_{21}\right)=\text{U}\left(C_{22}\right). \end{array}$$
  The immediate question now is: how can α be elicited? We will address this question in due course in section 3.3. For the time being, we only retain the important insight that, while the expected utility of an identification decision depends on (i.e., corresponds to) the probability that the person of interest is the source of the crime stain (proposition θ_1_), the expected utility of an inconclusive decision is a *constant*; i.e., it does not depend on the probabilities of the propositions θ_1_ and θ_2_. Instead, it corresponds to the utilities U(*C*_21_) and U(*C*_22_), which are taken to be equal.
- *Exclusion*: Here we focus on the following two consequences: (1) *C*_31_, the consequence of concluding “exclusion” (*d*_3_) when in fact the person of interest is the source of the crime stain (θ_1_), and (2) *C*_32_, the consequence of concluding “exclusion” (*d*_3_) in the event that an unknown person is the source (θ_2_). The latter, *C*_32_, is an accurate conclusion and we can assign to it the utility 1, expressing the idea that it is as desirable as an accurate identification (see consequence *C*_11_ above). A wrong exclusion, *C*_31_, is an inaccurate determination, and we should thus assign it a low utility to express that it is an undesirable outcome. One can ask, however, whether a false exclusion is as undesirable as a wrong identification (consequence *C*_12_). For the purpose of this discussion, let us assume that we consider the utility of a wrong exclusion, U(*C*_31_), to be equal to zero. The expected utility of decision *d*_3_ (exclusion) then is:
  $$\begin{array}{l} \text{EU}\left(d_{3}\right)=\underset{0}{\underset{︸}{\text{U}\left(C_{31}\right)}}\mathrm{Pr}\left(\theta_{1}|I\right)+\underset{1}{\underset{︸}{\text{U}\left(C_{32}\right)}}\mathrm{Pr}\left(\theta_{2}|I\right)\\ \;=\mathrm{Pr}\left(\theta_{2}|I\right). \end{array}$$
  As can be seen, since Pr(θ_2_∣*I*) = 1−Pr(θ_1_∣*I*), the expected utility of an “exclusion” is one minus the expected utility of an “identification.” The latter was found to be equal to Pr(θ_1_∣*I*), the probability that the person of interest is the source, whereas the former is found to be equal to Pr(θ_2_∣*I*), the probability that an unknown person is the source. Note that this result, the expected utility of an exclusion being directly proportional to the probability of the alternative proposition (θ_2_), follows from the assumption that a wrong exclusion has a utility of zero, U(*C*_31_) = 0, hence offering a symmetry between decisions. The reader is free to consider a different ranking, for example one where a wrong exclusion is felt to be less undesirable than a wrong identification, hence to assume a value for U(*C*_31_) greater than zero^7^. However, it may be easily shown that, for values close to zero, the expected utility EU(*d*_3_) for exclusions will correspond, approximately, to Pr(θ_2_∣*I*).

It follows from the above considerations that, despite the seemingly technical arguments involved, the expected utilities of the three decisions of interest reduce to rather basic terms: in case of the conclusions “identification” and “exclusion,” the modest assumptions made (e.g., the 0−1 scale, the particular ranking of preferences and the choice of the reference consequences) imply that the expected utilities are values given by the probabilities for the main propositions θ_1_ and θ_2_, respectively (i.e., the person of interest or an unknown person being the source of the crime stain). The typical objection is that these numbers are “unknown”^8^ to the decision-maker. However, it must be acknowledged that they reflect the decision-maker's level of uncertainty, given all the relevant information available at the time that the decision needs to be made. Therefore, these probabilities are, in principle, available to the decision-maker. So, a utility elicitation is not really needed for consequences of decisions *d*_1_ (identification) and *d*_3_ (exclusion). In turn, the expected utility of inconclusive decisions is a constant that corresponds to the utility assigned to the consequences of inconclusive decisions irrespective of which states of nature, θ_1_ and θ_2_, is true. Eliciting this utility requires further considerations (see below in section 3.3). Note that, whenever a different ranking is considered (e.g., considering a false exclusion to be a less severe outcome than a false identification), the elicitation process will be slightly more demanding. Further elaboration of this is given in Biedermann et al. (2008).

These considerations do not tell us yet how adequate (or inadequate) an inconclusive decision is, and how such a conclusion compares to rival decisions, such as an “identification.” As is clear from the above development, the utility of the consequences of reporting “inconclusive” is the pivotal issue. We will consider this question in the next section.

### 3.3. Clarifying Utility Elicitation for Consequences of “Inconclusives” and Determining Optimal Decisions

**Figure 1:**
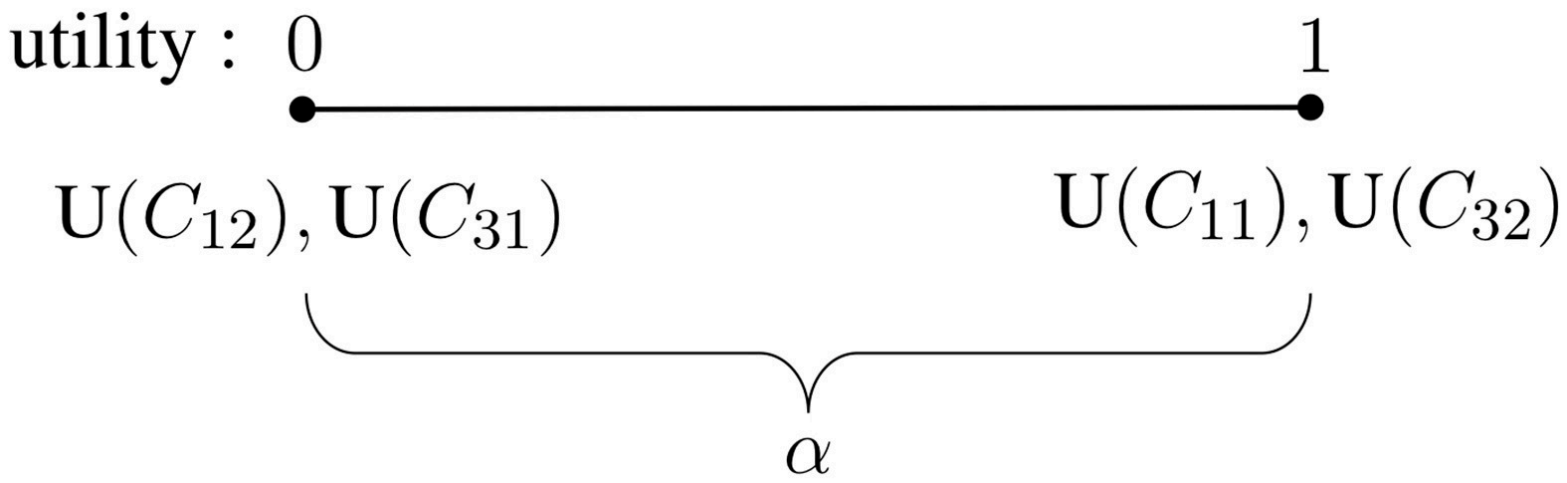
Representation of the utilities for the best decision consequences, correct identification (*C*_11_) and correct exclusion (*C*_32_), assigned as 1, and the worst consequences, false identification (*C*_12_) and false exclusion (*C*_31_), assigned as zero. The utility that remains to be elicited is α, the value assigned for the consequences of reporting “inconclusive,” *C*_21_ and *C*_22_. It can take any value in the range between 0 and 1 (including these endpoints).

Eliciting a value for α, the utility of the consequences of reporting “inconclusive” (*C*_31_ and *C*_32_), amounts to locating α on the range of utilities between 0 and 1, keeping in mind that these values have been assigned to, respectively, the worst and the best consequences (see also section 3.2 and summary in Figure 1). This utility assignment—which will directly determine the expected utility of the decision to report “inconclusive” (following the considerations given in section 3.2)—is meaningful in the sense that, if we consider the decision consequences of “inconclusives” of value, their utility should tend toward the utilities of the best decision consequences; in turn, if we consider “inconclusives” of low value, the assigned utility should tend toward those assigned to the worst decision consequences. The tricky issue is, however, to elicit this key utility (α) in a transparent way.

**Figure 2:**
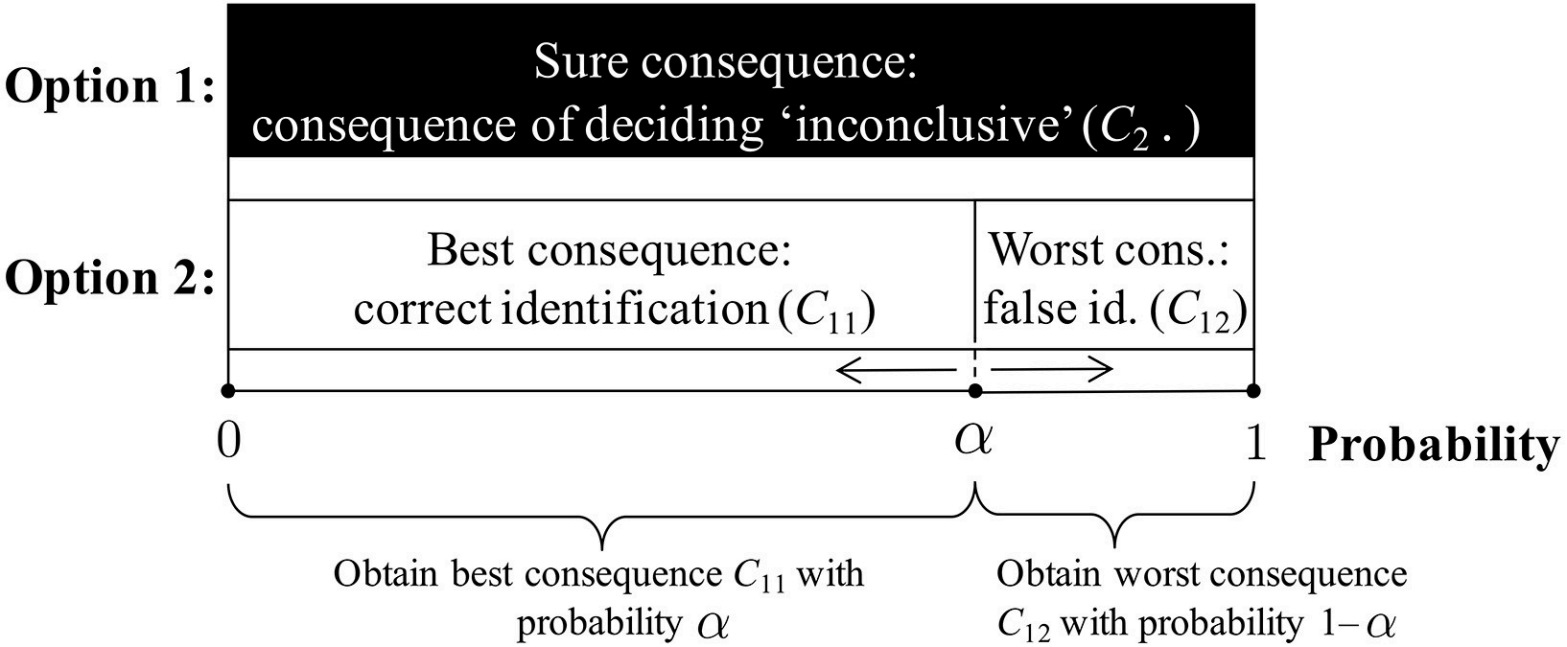
Illustration of the utility elicitation procedure. Option 1 involves having the consequence of deciding “inconclusive” for sure. Option 2 involves having the best consequence (correct identification, *C*_11_) with probability α and the worst consequence (false identification, *C*_12_) with probability 1−α. The procedure asks one to specify the probability that would make one indifferent between option 1 and option 2. This value then corresponds to the utility of the consequence for which a value needs to be elicited (here, the consequence of deciding “inconclusive,” *C*_2·_). Note that the particular value α shown in the figure is an example. The value α can vary in both directions toward 0 and 1, as illustrated by the edges ← and →.

To overcome this challenge, we can invoke a standard device for utility elicitation (De Groot, 1970) that involves the comparison of, on the one hand, the consequence for which a utility needs to be assigned (here, the consequence of deciding “inconclusive”), and on the other hand, two reference consequences for which utilities are available. We choose these reference consequences to be a correct identification (consequence *C*_11_) and a false identification (consequence *C*_12_), i.e., examples of the best and worst consequence, respectively. The consequence of an inconclusive decision thus is intermediate with respect to these two reference consequences. The procedure for utility elicitation, summarized in Figure 2, then works as follows^9^:

- Imagine you have two options. The first option is to have—for sure—the intermediate consequence, *C*_2·_ (i.e., either *C*_21_ or *C*_22_), the result of an inconclusive decision. The second option is a situation in which you may incur the best consequence, i.e., a correct identification (*C*_11_), with probability α, and the worst consequence, i.e., a false identification (*C*_12_) with probability 1−α.
- Next, ask yourself what would be the probability α in the second option that would make you indifferent between options 1 and 2. Stated otherwise, you need to give the probability α with which the second option would give you the best consequence (and with probability 1−α the worst consequence) so that you would be prepared to exchange it with option 1 (which implies the intermediate consequence *C*_2·_ for sure).
- It can be shown (De Groot, 1970) that the utility for the intermediate consequences can be found, under modest conditions, as
  $$\alpha \text{U}\left(C_{11}\right)+\left(1-\alpha \right)\text{U}\left(C_{12}\right)=\alpha .$$
  The probability α in option 2 that makes it equivalent, for you, to option 1 then is *your* utility for the intermediate consequence (i.e., the consequence of deciding inconclusive, *C*_2·_).

One way to look at this utility elicitation procedure is to focus on the following key question: how *low* should the probability of incurring the worst consequence in option 2 be in order to make it “viable” for the decision-maker, i.e., so that option 2 appears as acceptable as option 1 (in which the decision-maker would obtain the consequence of deciding “inconclusive” for sure)? Under the generally accepted view that wrongly identifying a person of interest is a consequence that ought to be avoided, this probability should be low, or even very low. Accordingly, the probability of obtaining the best consequence in option 2 will be high, or very high. But this probability—in our procedure—corresponds to the utility of the consequence of deciding “inconclusive”; and, thus, will be (very) high, too.

As we have seen, consequences of “inconclusive” decisions are *not* as inadequate as one may think they are. In particular, if the requirement is to make up one's mind seriously and justify a coherent ordering of decision consequences, which involves the proper appreciation of both highly desirable and undesirable decision consequences, the utility of consequences of inconclusive decisions can actually be found to be (very) high—unless one holds unusual preferences such as not considering a wrong identification as undesirable. This analysis also raises critical questions, however, and we discuss them further in the next section.

## 4. Discussion and Conclusions

### 4.1. Objections

A possible objection to the result of the previous section is that terms such as “low” or “very low” (associated to probabilities or utilities) are undefined. Also, it may be argued that questions such as “how *low* should the probability of incurring the worst consequence in option 2 be (…)?” (section 3.3 and Figure 2), cannot be answered in principle because they require a numerical precision that, in practice, most importantly in real casework, cannot be achieved. But this does not imply that the development as such is inapplicable or of no value. The value of the conceptual framework consists in helping to scrutinize and understand the logical properties of our qualitative and verbal assertions, and to critically examine intuitive judgments. Take, for example, the following statement, that follows from our conceptual framework:

*The assessment of the utility of the consequence of an inconclusive decision is tied—i.e., inversely proportional—to the probability of incurring the worst consequence that the decision-maker considers acceptable*.

Precise numbers are not necessary to see how the underpinning concepts in this statement relate to each other. However desirable or undesirable one *intuitively* judges the consequence of an inconclusive decision (i.e., without a check against a logical analysis of the problem), our framework tells us that, in a qualitative sense, *the smaller the probability of a wrong identification (in option 2;* *Figure 2**), the* higher *one's utility for the consequence of an inconclusive decision*. Thus, a person cannot be averse to a wrong identification *and* consider the consequence of an inconclusive decision as having a low utility *without* being incoherent. This is one example of what we mean by scrutinizing the logical properties of (intuitive) judgments that commentators may maintain.

As regards the issue of numeracy, the above qualitative level of analysis is the minimum to which one needs to commit oneself in order to issue meaningful statements, for no helpful guidance can be conveyed without at least a qualitative statement about how distinct assessments compare and relate to each other. This view is not new. It has previously been raised in defense of the use of probabilistic reasoning. For example, Friedman has noted: “(…) the theory need not be applied in its most powerful gear. On the contrary, it is a flexible template. It can take into account as much complexity as its user is able to handle.” (Friedman, 1997, p. 288) More recently, the same author has noted:

“(…) the concept of *magnitude* is essential. It is not enough to say that the harm caused by an incorrect choice of [decision] one is greater than that caused by an incorrect choice of [decision] two; to set the standard of persuasion appropriately, we need to have a sense of *how much* worse one error is than the other.” (Friedman, 2018, p. 2).

Critics may find this guidance unhelpful and argue that, at the end of the day, they still do not know how much worse one consequence ought to be judged than another. However, such an objection merely shows that the kind of question that needs to be addressed is not a scientific one, as it goes beyond the scope of the forensic practitioner's area of competence (e.g., Stoney, 2012).

### 4.2. Summary: Clarifying the Notion of “Inconclusive” Decisions

Inconclusive decisions have recently attracted skeptical comments in forensic science literature. For example, Dror and Langenburg refer to them as a “broad and imprecise decision category for fingerprint examiners” (Dror and Langenburg, 2018, p. 5) and distinguish them from what they call a “definitive categorical decision (i.e., identification or exclusion …)” (Dror and Langenburg, 2018, p. 5). We have argued throughout this paper, using a formal analysis, that inconclusive decisions are not “imprecise,” but can be given a precise definition which is as clear as the definitions of the rival decisions “identification” and “exclusion.” Stated otherwise, “inconclusive” decisions are *no less* “definite” than identification or exclusion decisions.

The results of the analysis and related effects considered in this paper may not be intuitively obvious. Moreover, it must be emphasized that the discussed decisional problem has been simplified for the sake of illustration. This means that, in practice, a decision-maker may need to handle situations where the answer is even less obvious. It is important, therefore, that discussions about inconclusive decisions be clear about the way in which the key elements of a decision problem, such as decisions, states of nature and consequences are defined, and how the desirability/undesirability of incurred outcomes can be measured. Moreover, as our development shows, intuitive approaches to the notion of “inconclusive” decisions may be liable to fallacious or counterintuitive claims: for example, it follows from our analyses that inconclusive decisions are *not* as inadequate as they are generally said to be – though there can be situations in which they do not represent the *optimal* course of action (i.e., reporting conclusion). Below, we will restate this result and summarize some of the other accompanying insights.

**Figure 3:**
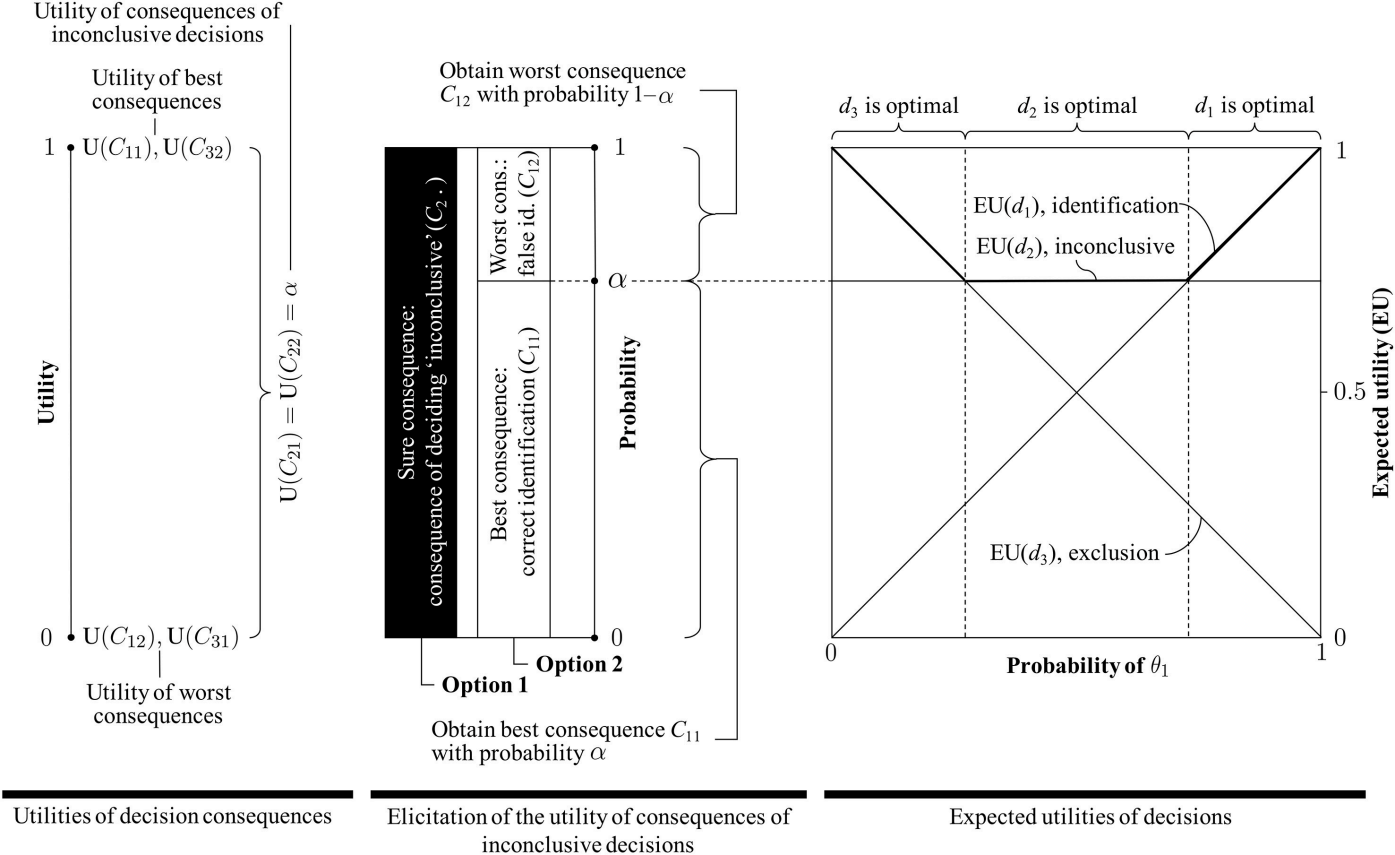
**(Left)** Definition of the (0, 1) utility scale and assignment of utilities of 1 to the best decision consequences (i.e., correct identification, *C*_11_, and correct exclusion, *C*_31_) and utilities of 0 to the worst consequences (i.e., false identification, *C*_12_, and false exclusion, *C*_31_). The utility α of the consequences of inconclusive decisions can take any value between 0 and 1, including these endpoints. **(Middle)** Elicitation of the utility for consequences of inconclusive decisions based on a comparison between two options. The utility of an inconclusive decision is obtained as the probability α with which Option 2 leads to the best consequence (and with probability (1−α) to the worst consequence), such that Option 2 is as desirable as Option 1 (which offers the consequence of an inconclusive decision for sure). **(Right)** Representation of the expected utilities of decisions “identification” (*d*_1_), “inconclusive” (*d*_2_) and “exclusion” (*d*_3_) (the bold line indicates the decision with the highest expected utility). The vertical dashed lines indicate transition points, i.e., where one decision becomes preferable to another decision (i.e., where one decision has a higher expected utility than another). Note, in particular, that the smaller the acceptable probability (1−α) for a false identification (*C*_12_) elicited in the procedure illustrated in the Figure in the middle, the closer to 1 the expected utility of the decision “inconclusive” in the Figure on the right, and hence the smaller the ranges of probabilities for θ_1_ (i.e., the proposition according to which the person of interest is the source) for which either “identification” or “exclusion” will be the optimal decisions (i.e., decisions with the highest expected utility). In the extreme case, when the acceptable probability (1−α) for incurring a false identification is zero, the expected utility of the decision “inconclusive” is 1, and hence maximal, in all cases (i.e., regardless of the probability of θ_1_).

1. First and foremost, it is important to note that the term “utility” qualifies the desirability/undesirability of decision *consequences*, such as the outcome (i.e., consequence) of deciding “identification” when in fact the person of interest is the source of the crime stain. This is an example of an accurate decision outcome, and generally regarded as desirable, yet the decision “identification” itself does *not* have an utility. Because it is not known, at the time of making the decision, which state of nature actually holds (i.e., whether the person of interest or an unknown person is the source of the stain), one can—at best—consider the *expected utility* of a decision. The expected value of a decision is obtained by weighing the utilities of the possible decision consequences by their respective probability of occurrence: for example, the utility of a correct identification will be discounted by the probability of obtaining it. Different rival decisions can be characterized and compared based on their expected utility; the idea being that a decision is considered “better” that another if its expected value is greater than that of the other.
2. Obtaining the expected utility of a decision may involve tedious steps, such as the elicitation of the utility function. However, it has been shown in our application to forensic identification that under modest and entirely reasonable assumptions (see section 3.2), the number of values that will need to be elicited is small. The expected utilities of all three decisions of interest reduce to clearly recognizable forms (see also on the right in Figure 3):
  - *Identification*: The expected utility of an identification corresponds (i.e., is directly proportional) to the decision-maker's probability that the person of interest, rather than an unknown person, is the source of the crime stain: EU(*d*_1_) = Pr(θ_1_∣*I*), shown as an ascending straight line.
  - *Exclusion*: The expected utility of an exclusion is *inversely proportional* to the decision-maker's probability that the person of interest, rather than an unknown person, is the source of the crime stain: EU(*d*_3_) = 1−Pr(θ_1_∣*I*), depicted as a descending straight line.
  - *Inconclusive*: The expected utility of an inconclusive decision is a constant (denoted here α), shown as a straight horizontal line. Following the elicitation procedure introduced in section 3.3 (summarized in the middle of Figure 3), this utility is related to the decision-makers' aversion for a false identification.
  In summary, thus, the utilities of the consequences of all three rival decisions are *fixed*. The expected utilities of the decisions, however, *vary* as a function of the probability that the person of interest, rather than an unknown person, is the source of the crime stain (proposition θ), *except* for the decision “inconclusive”: its expected utility is equal to the utility of the related decision consequences and is *not* influenced by the uncertainty about the state of nature θ^10^.
3. Regarding the particular case of inconclusive decisions, we note that, as the decision-maker's probability (1−α) associated to a false identification (in option 2; Figure 2) decreases (i.e., tends to zero), the utility for the consequences of inconclusive decisions increases (i.e., tends toward one). Stated otherwise, a decision-maker who will only consider acceptable to make a false identification (in option 2; Figure 2) with a low (or very low) probability (1−α) will maintain a high utility for the consequence of an inconclusive decision. In the extreme case in which the acceptable probability for a false identification is zero, the decision-maker:
  - considers the consequence of an inconclusive decision as desirable as an accurate conclusion, i.e., a correct identification or exclusion, expressed in terms of an utility of 1, and
  - considers an inconclusive decision as optimal in all situations. As seen in Figure 3, for (1−α) = 0, the expected utility of an inconclusive decision takes the value 1, and no other decision (i.e., identification or exclusion) has a higher expected utility across the entire scope of probabilities for states of nature θ. Moreover, an inconclusive decision will have a higher expected utility than all other decisions except for the special cases where Pr(θ_1_∣*I*) is either zero or one. In the former case, both the expected utility of an exclusion and of an inconclusive decision are 1; in the latter case, both the expected utility of an identification and an inconclusive decision take the value 1.

Finally, note also that the formal criterion for singling out optimal decisions used in this paper should be understood as a conditional advice (Biedermann et al., 2018), the scheme does not *prescribe* particular decisions. It is perfectly conceivable that a decision maker may decide to report “inconclusive” even though—according to the formal analysis—an identification may be the optimal decision (i.e., the decision with the maximum expected utility). This does not necessarily represent a problem, but a situation that would merit further inquiry, including a review of one's evaluation of undesirability of adverse consequences. Indeed, there may be various circumstantial (and legitimate) reasons and personal motives that drive a person to act in a way that is suboptimal with respect to the recommendations following the maximum expected utility criterion. For example, the decision-maker may wish to avoid the overall worst consequence (i.e., a false identification) at all costs, a goal that can only be achieved by never selecting the identification decision *d*_1_ (Biedermann and Vuille, 2018). See also Dror and Langenburg (2018) further discussion about the justification, and possible lack thereof, of inconclusive decisions.

### 4.3. Scrutinizing General Claims and Decision Policies

Throughout this paper, we have argued that the decision-analytic approach does not seek to impose a numerical precision to all practical arguments if the discussants are not willing to engage with it. Instead, we have emphasized that the merit of the approach consists in scrutinizing the logical foundations of general claims and opinions commonly raised by practitioners and researchers in the field of forensic identification. The particular claim we have looked at, shown to be unreasonable and refuted, is to say that inconclusive decisions are inadequate, vague and undefined. Specifically, we have shown that, unless the decision-maker shows little concern about false identifications, inconclusive decisions not only have

- a high expected utility (i.e., close to 1), but
- also represent the most preferable conclusion in a predominant number of the possible cases (i.e., cases differing by their probability for the proposition that the person of interest is the source).

To conclude, we illustrate our point with one last observation. Consider the claim that “inconclusive” decisions, because of their alleged drawbacks, should disappear, and a given expert would always have to report either an “identification” or an “exclusion.” Would such a conclusion policy make sense? What would it imply, conceptually? We can investigate these questions by studying the plot shown on the right in Figure 3. As is readily seen, in order for the inconclusive decision to have a lower expected utility than either the decision “identification” or “exclusion,” one needs to assume a higher probability (1−α) for incurring a false identification. Specifically, for the case depicted in Figure 3 (right), and the assumptions made in Section 3.3, the utility of the consequence of an inconclusive decision would need to be lower than 0.5. However, this would also mean that the decision-maker would be considerably less averse to a false identification. As the utility elicitation procedure shows (Figure 3, middle), assessing α < 0.5 would mean that the decision-maker is prepared to “exchange” the consequence of an inconclusive decision (Option 1) with an option which leads to a false identification with probability (1−α), which is *greater* than 0.5 (i.e., Option 2). It is difficult to imagine any criminal case in which such a preference structure would be defensible.

Although our analyses in this paper highlight valuable features of inconclusive decisions, we do not intend to convey the idea that this type of conclusion, or any other type of conclusion in the IIE paradigm, is suitable or recommendable for forensic scientists. In fact, as has been emphasized, *deciding* what to conclude requires value judgments (i.e., utilities/losses) that are widely considered to be beyond the scientist's area of competence (e.g., Champod et al., 2016). Instead it is more suitable and defensible for forensic scientists to limit their reporting to their observations and findings only, and their associated probative value (e.g., Willis et al., 2015).

## Author Contributions

AB is the primary author of this manuscript. All authors made equal and substantial contributions to the analyses and exchanges that are at the basis of this original research paper.

### Conflict of Interest Statement

The authors declare that the research was conducted in the absence of any commercial or financial relationships that could be construed as a potential conflict of interest.

## Footnotes

^1^See, for example, the discussion by Stoney (1991) on the fundamentally different approaches to interpretative reasoning on fingermark evidence as compared to DNA.

^2^Note that throughout this paper, we will refer to conclusions provided by forensic examiners as *decisions* in the sense that the choice of one among different conclusions, such as IIE, is considered to be a decision (Biedermann et al., 2008, 2016). This has a direct impact on the formal methods of analysis that we will rely upon in this paper (see also section 2).

^3^Readers well acquainted with decision theory may skip this section.

^4^In the present paper, we will consider only the situations where the consequences *C*_*ij*_ as a result of a combination between a decision *d*_*i*_ and a state of nature θ_*j*_ are certain. Cases where there is uncertainty about the consequences are not covered.

^5^Note that there may be more than one “best” (“worst”) outcome. For example, one may consider a correct exclusion equally desirable as a correct identification.

^6^The probability of occurrence of a given consequence depends on the probability of the given state of nature, denoted here by θ. Generally, we condition probabilities by the relevant background knowledge, denoted *I*. Hence, we write the probability that the person of interest is the source of the mark, given the available information, as Pr(θ_1_∣*I*).

^7^See Biedermann et al. (2008) for examples and graphical illustrations of situations in which U(*C*_31_) is considered different from zero.

^8^See Biedermann (2015) for a review of historical sources on why assertions of non-knowledge of probabilities (i.e., “unknown probabilities”) are nonsensical.

^9^See Biedermann et al. (2008, 2016) and Taroni et al. (2010) for further details on the mathematical background of the procedure.

^10^Note again that these properties and observations follow from the preference structure and assumptions outlined in section 3.2.
